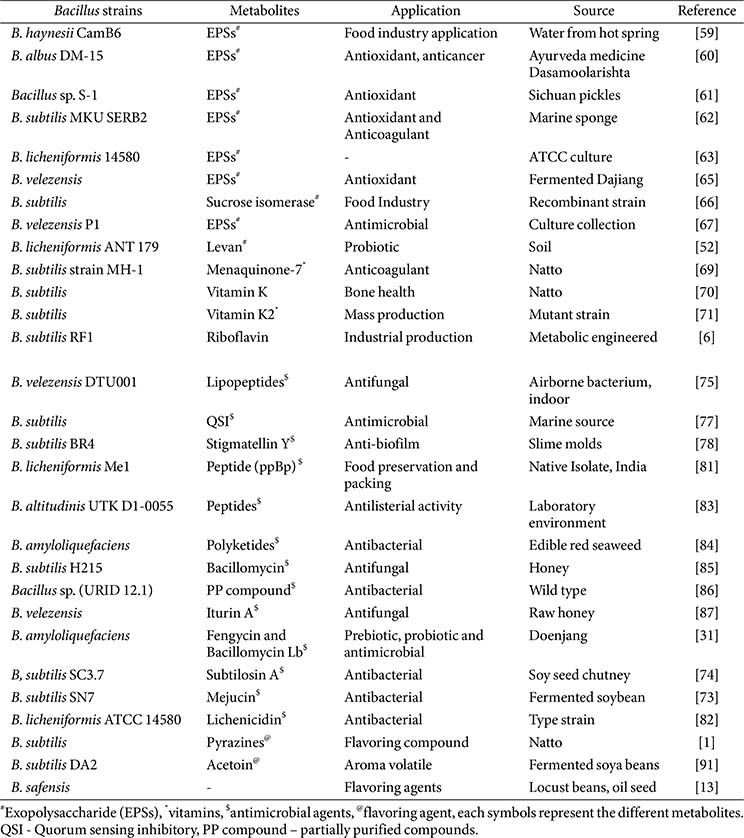# Role of *Bacillus* Species in Food Industry: Advantages and Limitations

**DOI:** 10.4014/jmb.2507.07043

**Published:** 2025-10-22

**Authors:** Jegadeesh Raman, Jun Su Noh, Jeong-Seon Kim, Gyeongjun Cho, Do-Hyun Kim, Jaekyung Song, Soo-Jin Kim

**Affiliations:** Agricultural Microbiology Division, National Institute of Agricultural Sciences, Rural Development Administration, Wanju-Gun, Jeollabuk-Do 55365, Republic of Korea

**Keywords:** *Bacillus*, food industry, fermentation, probiotics, metabolites, flavor

## Abstract

*Bacillus* species have played a significant role in food safety and production throughout history. While some of these species have been widely used in culinary applications and fermentation, many lack scientific evidence to support their use. Integrating the *Bacillus* species into fermented food is a longstanding culinary tradition that reflects deeply rooted cultural customs and regional expertise. In traditional cuisines, these species contribute beneficial metabolites and distinctive flavors, adding a unique cultural dimension to the food. Asian fermented foods containing *Bacillus* species are recognized for their probiotic properties, which enhance food safety by preventing foodborne infections. *Bacillus* spores, such as *B. subtilis*, act as probiotics, although their mechanisms of action are not yet fully understood. Understanding the different *Bacillus* species is crucial for determining whether they function as probiotics or pathogens. The use of *Bacillus* in pharmaceuticals has positive effects on human health. Fermented products increase immunity, reduce bone loss, and alleviate allergies, thereby supporting the safety of the food and animal sector. *Bacillus* probiotics are incorporated into food products and supplements due to their health benefits. Additionally, *Bacillus* species are ecologically important for their ability to produce various enzymes and break down organic matter in the environment. Industrially, they have numerous applications, including synthesizing antibiotics, lipopeptides, extracellular polysaccharides, flavoring agents, and substantial quantities of proteins and enzymes. The *Bacillus*-fermented food sector is growing due to increased demand for non-dairy functional products. Progress in strain validation and regulation enables safe distinction between beneficial and harmful species.

## Introduction

*Bacillus* is one of the most ancient and diverse genera within the Bacillaceae family. These gram-positive, rod-shaped bacteria are known for their widespread distribution. *Bacillus* species can survive in various habitats, including soil, water, and plant surfaces. Their remarkable ability to form stress-resistant endospores and resist adverse environmental conditions makes them highly adaptable. This genus is of significant industrial importance due to its rapid proliferation, short fermentation cycles, and ability to secrete substantial quantities of extracellular enzymes. Notably, it produces lipases, carbohydrate-active enzymes, and proteases, which are widely employed in the food industry, animal feed production, and other biotechnological applications [1, 2]. *Bacillus* species are considered promising probiotics, offering diverse advantages for gastrointestinal health, immunological response, and bone health. *Bacillus* species are much desired as nutraceutical formulations that promote overall health in the global market. However, the nutraceuticals market has yet to accept commercial products that broadly use *Bacillus* for functional foods. This hesitancy may stem from ongoing debate over the safety classification of *Bacillus* species. Hence, it is crucial to understand the phenotypic and genotypic traits of particular *Bacillus* species and their validation with those possessing GRAS “Generally Recognized as Safe” status.

The FDA has classified *B. amyloliquefaciens*, *B. atrophaeus*, *B. clausii*, *B. coagulans*, *B. licheniformis*, and *B. subtilis* as GRAS [3]. Among them, the *B. subtilis* species have extensively undergone fermentation processes for Southeast Asian chungkookjang and natto, as well as in West African products such as gari and dawadawa. These fermented foods are distributed across regions including Korea, Japan, Nepal, Ghana, Nigeria, and Burkina Faso. Despite their similar preparation process and appearance, these foods are known by different names in each country. Worldwide, various fermented foods exist, including products made with *Bacillus*. Bamboo shoots, cassava, and legume-based fermented foods incorporate *Bacillus* species [4]. Recent research has emphasized the need to characterize *Bacillus*-fermented foods (BFFs), specifically those produced in rural households, which remain underexplored compared to commercial counterparts. Genomic and metabolomics analyses of strains from Asian fermented foods are being used to design improved starter cultures and validate health-related properties [5-7]. In parallel, studies from West Africa have focused on isolating bacteriocin-producing *Bacillus* strains to combat *B. cereus* contamination, thereby improving food safety and broadening applications. These findings expand the utilization of *Bacillus* species in food applications and enhance their usage. Numerous *in vivo* and *in vitro* studies have investigated the metabolites of *Bacillus*, exploring their flavor-enhancing and health-promoting properties. Given the growing interest in nutritious food containing health-enhancing elements and the specific needs of young consumers, probiotic chocolate is a compelling product. Milk chocolate with *B. coagulans* is a distinctive and unconventional product in its category [8]. However, most probiotic products still focus on *Lactobacillus*. Notably, several *Bacillus*-containing fermented foods have received official recognition as probiotics, and *Bacillus*-based supplements are now marketed across Asia, Europe, and the U.S. This review provides novel insights into the probiotic potential of *Bacillus* species isolated from traditional fermented foods, which could inform the development of safer and more effective functional foods.

## *Bacillus* Species in the Food Industry

### Source of Beneficial *Bacillus* Species

**Fig. 1:**
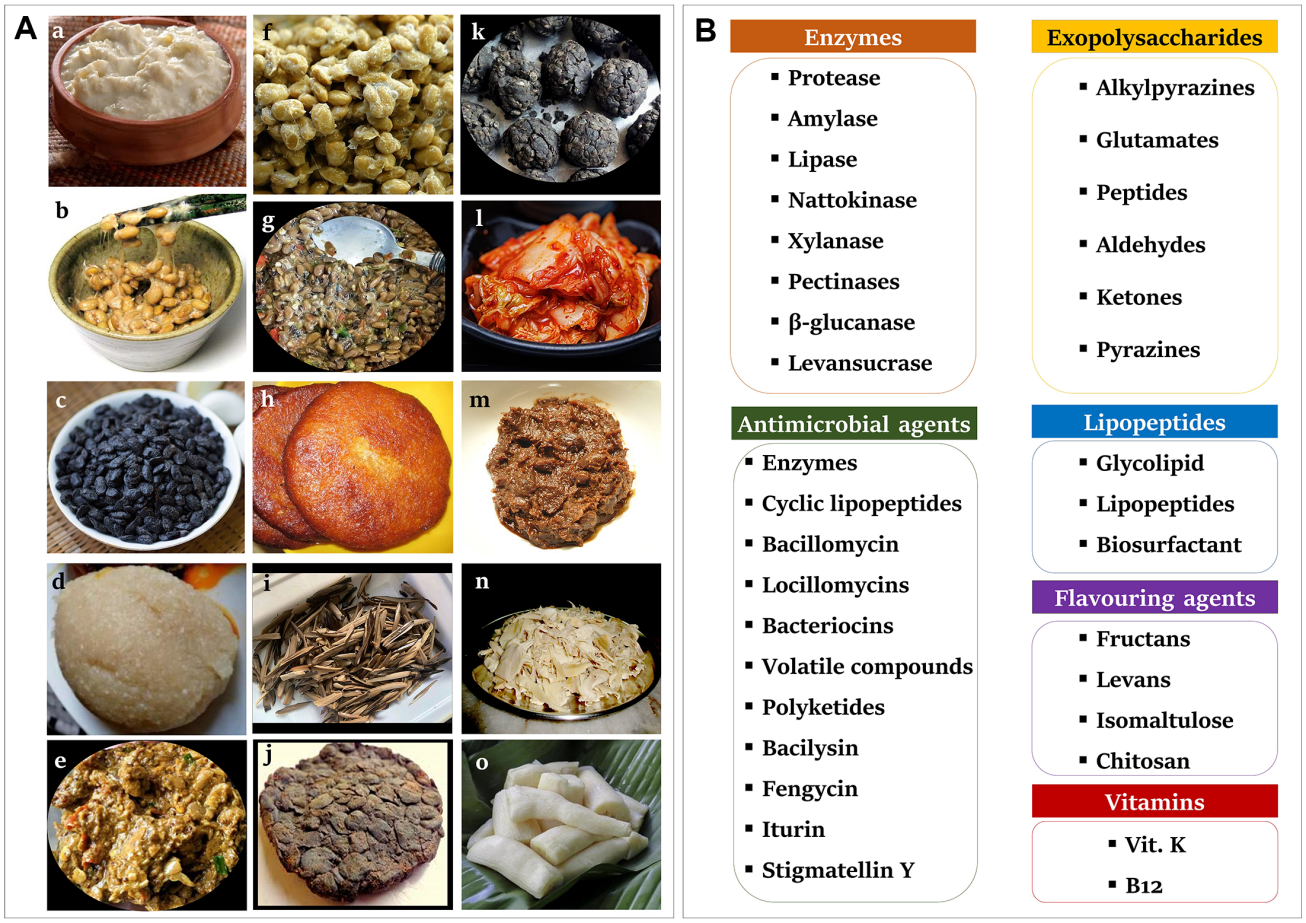
*Bacillus* indigenous fermented food and their bioactive metabolites. (**A**) Fermented food produced by the *Bacillus* species of indigenous origin (a) Rabadi (India), (b) Natto (Japan), (c) Douchi (China), (d) Gari (Africa), (e) Tungrymbai (India), (f) Cheonggukjang (Korea), (g) Bekang (India), (h) Adhirasam (India), (i) Ugba (Nigeria), (j) Iru (West Africa), (k) ogiri (West Africa), (l) Kimchi (Korea), (m) Doenjang (Korea), (n) Soibum (India), (o) Tapai Ubi kayu (Malaysia). Images are sourced from Wikimedia Commons and are available for free download. (**B**) Bioactive metabolites from *Bacillus* species (Enzymes, Exopolysaccharides, Antimicrobial agents, Lipopeptides, Flavoring agents and Vitamins).

The *Bacillus* and *Lactobacillus* species (LAB) demonstrate functional similarities, and both groups of species are prominent candidates for probiotic application. Besides, *Bacillus* species exhibit higher acid tolerance and enhanced stability during heat processing and low-temperature storage. The spores have high heat tolerance and can survive extremely harsh environmental conditions. The animal’s gastrointestinal tracts include numerous species of *Bacillus*, which may originate from plant material. *Bacillus* species, found in various sources, are frequently present in fermented foods [9]. Several species isolated from traditional fermented foods such as natto (Japan), soibum (India), chungkookjang (Republic of Korea), douchi (China), gari (Africa), tapai ubi kayu (Malaysia), rabadi (India, Pakistan), and ugba (Nigeria) are currently utilized in the industrial production of functional products (Fig. 1A) [10 -17]. Table 1 indicates the strain identity, source of isolation, and country of origin of indigenous fermented foods. Several *Bacillus* species have been isolated from tungrymbai and bekang, naturally fermented soybean foods from India. Among these bacterial isolates, the most dominant species was *B. subtilis* [14]. Phylogenetic investigations and 16S rDNA gene sequences have shown that most bacteria present in fermented foods are closely related to *Bacillus*, specifically *B. subtilis* subsp. *subtilis*, *B. tequilensis*, *B. siamensis*, and *B. safeness* [15]. However, many *Lactobacillus* and *Bacillus* species were obtained from the traditional Korean side dish, kimchi. Nevertheless, despite their crucial role in the food business, there is a need for more research addressing the succession or ontogeny of *Bacillus* species at the species level during kimchi fermentation, comparable to other environments [18]. The species, *B. subtilis* and *B. velezensis*, have gained significant interest in the food industry as valuable biological resources. Interestingly, *B. subtilis* var. natto, *B. clausii*, *B. licheniformis*, and *B. coagulans* are currently used worldwide to enhance the quality and popularity of functional foods. The fermentation process, which involves specific temperature ranges, pH levels, and increased aeration, promotes the growth of aerobic spore-forming bacteria belonging to the genus *Bacillus*. These species consist of *B. pumilus*, *B. licheniformis*, and *B. subtilis*. The commercially employed species include *B. subtilis*, *B. clausii*, *B. coagulans*, *B. licheniformis*, *B. polyfermenticus*, and *B. pumilus*. The taxonomic classification of the genus *Bacillus* has experienced significant alterations over time. Molecular taxonomy has led to the classification of *Bacillus* species into five distinct phylogenetic groupings [19].

### *Bacillus* Species in Food Fermentation

The microbial fermentation process increases and enhances the shelf life of foods, improving their quality and flavor. Additionally, *Bacillus* species are being investigated for their potential as probiotics, offering health benefits when consumed in sufficient quantities (Table 2). These species frequently generate fermented food items, including cheeses, fermented vegetables, and soy-based products [20]. Each subspecies of *B. subtilis* exhibits distinct biological characteristics due to variations in metabolite production. Therefore, the addition of red pepper powder to kimchi is expected to impact the development of *B. subtilis*, *B. velezensis*, and LAB species during the fermentation process [21]. Similarly, adhirasam, a traditional sweet from Southern India, boasts a distinct texture, flavor, and taste, primarily attributed to the starter cultures that make it a special dish. *Bacillus* species predominate, influencing the fermentation of the dough and significantly contributing to the products essence and structural characteristics [11]. A study examined the changes in the bacterial community over time during the production of traditional Nigerian fermented sauces, iru and ogiri, which are crucial condiments used to enhance the flavor of dishes and act as protein alternatives in the diets of rural communities throughout West Africa [13]. Notably, *B. velezensis* and *B. amyloliquefaciens* fermentation enhances the quality of bread. The breakdown of rigid hemicellulose in wheat fiber by *Bacillus* species improves bread quality. Additionally, sourdough fermentation and the use of microorganisms effectively control ropy spoilage [22]. The fermented substrate with *Bacillus* was identified as having a higher concentration of organic and volatile components. Furthermore, microbial fermentation can significantly enhance the nutritional quality and bioactivity of mixed feeds made from soybean meal and maize bran. The specific microorganisms involved in process, including *B. subtilis*, *B. pumilus*, and *Lactobacillus* species, play a crucial role in enhancing the overall quality of the feed [23]. Doenjang is a classic Korean condiment produced through the fermentation of soybeans and brine. It has garnered significant recognition as a nutritious source and flavor enhancer, offering a range of essential nutrients, including amino acids, flavonoids, vitamins, and minerals. Variety of *Bacillus* species have been found in Doenjang samples during fermentation. Whereas *B. subtilis*, *B. amyloliquefaciens*, *B. siamensis*, *B. methylotrophicus*, and *B. licheniformis* have significant roles in the fermentation process [23, 24]. *B. licheniformis* is used in conjunction with *Saccharomyces cerevisiae* to produce traditional fermented Korean food chungkookjang. Several studies have demonstrated the beneficial impact of *B. licheniformis* fermented food on the health of animals [25, 26]. Research has shown that diabetic rats with experimental Alzheimer's disease exhibited improved cognitive function and better glucose management when they were given *B. licheniformis* fermented soybeans [27]. In addition, *B. amyloliquefaciens* species are utilized in the food industry due to their broad range of applications. This strain is responsible for food fermentation and can produce antimicrobials, bioactive chemicals, extracellular polymeric substances, and peptides [28]. Furthermore, *B. amyloliquefaciens* plays a crucial role in synthesizing koji, which is essential for its manufacturing. The application of the strain results in notable changes in lipoproteins and flavonoid synthesis [29]. *Bacillus amyloliquefaciens*, despite having multiple strains, is an entirely safe and non-toxic microorganism in terms of both safety and toxin production. According to the FDA [30], it is generally considered safe for consumption and medical use. In addition, *B. amyloliquefaciens* HJ18-4 and RD7-7, obtained from Doenjang, a fermented Korean soybean paste, have been discovered to inhibit the harmful production of *B. cereus* in the food sector [31]. *Bacillus*-derived fermented products enhance the shelf life, quality, and flavor of food while also promoting the development of probiotics, functional foods, and improvements in animal feed. Additionally, they increase the levels of organic and volatile compounds, making diverse composite diets more nutritious and bioactive. This process is utilized across various industries to preserve the freshness of baked foods, create products from soybeans, produce health supplements, and generate antimicrobials and beneficial compounds. It employs GRAS strains such as *B. subtilis*, *B. licheniformis*, *B. coagulans* and *B. amyloliquefaciens*, which are crucial in the global food, feed, and beverage industries.

### Probiotic Properties of *Bacillus* Species

Probiotics are utilized in various sectors, including pet supplements, sports nutrition, nutritional supplements, and the food and beverage industry. The rising awareness of the health-promoting properties has substantially contributed to the expansion of this sector, which is anticipated to expand by 14% from 2023 to 2030, with a market value of USD 77 billion in 2022 [20]. *Bacillus* probiotics are more extensively promoted in Southeast Asia than in Western countries. On the other hand, there is a fast-growing interest in the United States and Europe [32]. The worldwide *Bacillus* probiotic market is expected to experience substantial growth in the upcoming years. The rise is primarily driven by the growing acceptance of the health benefits of *Bacillus* probiotics, particularly for maintaining gut health and supporting the immune system. *B. coagulans* spores are frequently used in probiotic products. It can withstand acidic conditions in the stomach and then travel to the intestines, thereby improving digestion, alleviating irritable bowel syndrome (IBS), and stimulating the immune system. The probiotic spore-forming bacteria can survive throughout cooking and storage, making them suitable for use in functionally prepared foods [33, 34]. The results also showed that the viability rate of *Bacillus* spores was higher than the minimum suggested daily therapeutic doses during sausage preparation and refrigerated storage [34, 35]. The bacterium *B. amyloliquefaciens* was isolated from the deep-sea shark and has been found to possess antibacterial and probiotic potential [36]. Furthermore, the strain obtained from soil samples showed beneficial effects for inflammatory bowel disease [37]. Larsen *et al*. (2014) found that *B. amyloliquefaciens*, *B. subtilis*, and *B. mojavensis* exhibit promising qualities as probiotics for pig feed [38]. However, *B. amyloliquefaciens* in broiler feed leads to enhanced growth and reduces the adverse effects of lipopolysaccharide on the immune system [39]. The spore-forming *B. subtilis* and *B. amyloliquefaciens* probiotics are utilized in animal husbandry to enhance growth and prevent disease. Supplementing soybean-derived byproduct animal food with probiotic species enhances its nutritional value, reduces manufacturing expenses, and minimizes adverse environmental impacts [40]. Interestingly, *B. amyloliquefaciens* has promising potential in eliminating mycotoxins and displaying probiotic capabilities [28]. On the other hand, *B. safensis* has proven beneficial for aquaculture, and researchers have observed that this strain enhances the health of fish [41]. The thermophilic strain *B. smithii*, obtained from various sources, shows great potential for application in the food industry. This strain is resilient to both gastric acid and high temperature, making it ideal for exhibiting probiotic advantages compared to typical commercial probiotics [42]. *Bacillus* species, known for their probiotic properties, exhibited superior survival compared to probiotic *Lactobacillus* species. The survival rate of *Bacillus* species with claimed probiotic traits is frequently above 83% [42]. The *Bacillus* species that demonstrate the stated probiotic qualities have been observed to possess the capability to survive within food matrices. According to Soares *et al*. (2019), *B. subtilis* and *B. coagulans* exhibit a prolonged shelf life, demonstrate stability, and resist simulated gastrointestinal conditions [43]. Cisowska *et al*. (2019) have developed a new type of probiotic chocolate that contains 357 active ingredients in concentrations equivalent to those found in existing products [8]. Overall, *Bacillus* probiotics are extensively used in food, supplements, and animal health due to their spore-forming durability. Species like *B. coagulans*, *B. subtilis*, and *B. clausii* can survive stomach acid, heat and storage, making them useful in functional food supplements. Compared to *Lactobacillus*, *Bacillus* probiotics offer great stability, a long shelf life, and better compatibility with different food products. Therefore, *Bacillus*-based probiotics are a robust and versatile option for the food and pharmaceutical sectors.

## Bioactive Metabolites from *Bacillus* Species

*Bacillus* species can produce a diverse range of metabolites that are widely used in commercial applications in agriculture, medicine, and biotechnology. In addition, *Bacillus*-derived enzymes are stable and effective for various applications, including food processing, fermentation, pharmaceuticals, and beverage production. Lipopeptides and antimicrobial metabolites act as natural preservatives, emulsifiers, and biocontrol agents in food, agriculture, and environmental settings. Functional foods, nutraceuticals, and biomedicines benefit from the inclusion of vitamins and exopolysaccharides (EPS). The taste of traditional and modern fermented foods is enhanced by flavoring compounds and volatile metabolites, which also support sustainable industrial practices. Fig. 1B shows a compilation of metabolites synthesized by *Bacillus* species. In addition, Tables 3 and 4 demonstrate the functional applications of metabolites derived from *Bacillus* in food processing, preservation, and their potential in the nutraceutical sector.

### Enzymes

*Bacillus* species have been shown to possess protease, amylase, lipase, and nattokinase. *B. amyloliquefaciens* SA35 and *B. subtilis* SB07, isolated from dry-cured sausages, grew under the optimized culture conditions and showed tremendous proteolytic and lipolytic activity during the ripening of the fermented sausages [44]. The *B. subtilis* strain PNG27 produced the milk-clotting enzymes, and sensory evaluation indicated that the cheese produced using these enzymes exhibited a better flavor and overall acceptability [45]. In recent years, the majority of commercial proteases have been derived from various *Bacillus* species, such as *B. clausii*, *B. licheniformis*, *B. amyloliquefaciens*, *B. pumilus*, and *B. gibsonii* [46]. Due to their wide variety of exceptional qualities, including high temperature and pH stability, they have been employed in the food industry to process various foods. Nattokinase (NK) is a serine protease obtained by purifying and extracting it from natto. NK is a powerful protein that effectively dissolves blood clots and is used to treat cardiovascular problems [11, 47]. Additionally, L-glutaminase production has been reported in *Bacillus* species, including *B. subtilis*, *B. amyloliquefaciens*, and *B. cereus*. Marine water isolates of *B. subtilis* produced L-glutaminase, and the enzyme exhibits potent antitumor activity against different cancer cell lines, sparing normal cells [48]. However, commercially essential enzymes, such as pectinases, hydrolyze pectic compounds linked by α-1, 4-glycosidic bonds and esterified with methyl groups. Pectinase enhances the absorption of nutrients from the substrate and contributes to microbial fermentation [49]. Notably, *B. subtilis* produced highly purified pectinase, in addition, *B. tequilensis* SALBT was found to be a good source of pectinases. The pectinase from this species has been proven effective in clarifying juice and in dehulling coffee beans [50]. The culture supernatant of *B. amyloliquefaciens* exhibiting xylanase activity facilitated the retrieval of approximately 43% xylose during brewer’s spent grain saccharification [51].

The enzyme levansucrase, obtained from the bacteria *B. licheniformis*, can hydrolyze sucrose into prebiotic fructooligosaccharides [52]. This enzyme exhibits thermotolerance and desiccation resistance. *B. licheniformis* is generally considered safe, making it ideal for levan production in food and biomedicine. Notably, recombinant enzymes can be produced in large quantities by genetically modified *Bacillus subtilis*, thereby meeting growing demands at low costs. The recombinant β-glucanase had a fermentation capacity of 242.02 Uml^-1^ h^-1^, and its concentration reached 4,840.4 Uml^-1^. These findings concluded that *B. subtilis* WB600 was a suitable host for expressing the 3-1,4-β-glucanase mutant, which has benefits for its use in the brewing industry [53]. Duarte *et al*.(2020) produce recombinant *B. amyloliquefaciens* transglutaminase gene (TGase) in *E. coli* using bicistronic vectors. The enzymes reticulating capacity is employed in food processing [5]. In addition, the recombinant strains of *B. cereus* in *B. subtilis* reduce the toxic level of acrylamide in food [54]. Wang *et al*. (2023) employed a customized approach that involved combining loop engineering and iterative saturation mutagenesis techniques to enhance protease activity under low-temperature environments [7].

### Lipopeptides

Microbes produce novel glycolipids and lipopeptide surfactants as functional ingredients in the food, processing, and pharmaceutical industries. Lipopeptides produced by *B. methylotrophicus* DCS1 exhibit significant *in vitro* antioxidant properties. Additionally, lipopeptides are used to protect oils and fats from oxidation in beef, as well as to enhance the preservation of ground beef patties [55]. Recent studies indicate that lipopeptides DCS1 are adequate for preserving fatty foods against lipid oxidation [55]. *Bacillus* species synthesize lipophilic, partially acetylated phenolic compounds derived from olive polyphenols, potent antioxidants important in the formulation of functional foods [56]. Surfactin is a cyclic lipopeptide produced by bacteria belonging to the genus *Bacillus*. Biosurfactants have several advantages over chemically synthesized surfactants, as they are not widely used commercially. *Bacillus* biosurfactants have demonstrated higher biodegradability, lower toxicity, greater thermostability, higher efficiency, longer storage time, and improved environmental compatibility compared to chemically synthesized surfactants. The biosurfactant properties of *Bacillus* can be utilized in the food industry, particularly as emulsifiers, solubilizers, antiadhesives, and antimicrobial agents (Table 3). Besides surfactin, there are also *Bacillus* lipopeptide families of iturins and fengycins, which share many properties similar to surfactin [57]. *Bacillus* sp. derived glycolipids have a wide range of applications, including enhanced oil recovery, food processing, and antimicrobial activity. Lipopeptides obtained from the endophytic *B. velezensis* FZ06, isolated from *Camellia assamica* leaves, have been found to inhibit food spoilage bacteria and toxic fungi in the food industry [58].

### *Bacillus* Exopolysaccharides

Exopolysaccharide synthesis is a natural process in probiotic bacteria, playing a crucial role in biofilm formation, bacterial adhesion, and bacterial cell aggregation, as well as water-holding ability, attracting nutrient sources, and providing a protective barrier. EPS synthesis in probiotics is essential for biofilm formation and other functions. Thermophilic *B. haynesii* produces it from an Andean hot spring, and the EPS functional properties are specifically as an antioxidant and for its emulsification, water-holding, oil-holding, and flocculation agent. The *Bacillus* EPS can be a valuable additive for the food-processing industry [59]. EPS is obtained from the probiotic bacterium *B. albus* which was isolated from the polyherbal, fermented traditional medicine Dasamoolarishta of Indian Ayurveda [60]. The production of EPS was significantly higher than previously reported for *Bacillus* species, such as *B. subtilis* (147.23 mg^-1^) and *Bacillus* sp. S-1 (35 mg l^-1^), which were isolated from marine sponges and fermented Sichuan pickles, respectively [61, 62]. Malick *et al*. (2017) found that *B. licheniformis* produces a high yield and high molecular weight EPS (48.57 g l^-1^) compared to *B. amyloliquefaciens* (6.7 g l^-1^) [63]. Notably, *B. subtilis* natto produces fructooligosaccharides and levans during fermentation, which are highly health-beneficial in food. Levans are commercially important fructose polymers, or fructans, used as a prebiotic fiber source [64]. *B. velezensis* isolated from fermented Da-Jiang, produced EPS throughout the bacterial growth period, with a yield of 2.7 of g l^-1^. The crude EPS isolated from *B. velezensis* exhibits antioxidant and probiotic properties, and it could be applied in the food industry [65]. An engineered *B. subtilis* strain that enables antibiotic-free and efficient production of sucrose isomerase using the CRISPR/Cas9 system and is used in the food industry [66]. *Bacillus velezensis* has been documented to produce EPS, lipopeptides, and siderophores, which enable it to combat pathogenic rice fungi such as *Cochliobolus lunatus* and *Magnaporthe oryzae* [67]. Additionally, measures to minimize bacterial and fungal contamination should improve mango fruit protection [68]. Binmad *et al*. (2022) reported that an exopolymeric material from *B. velezensis* P1 was introduced to chitosan made from squid pen. Mangoes were coated with a glossy, silky layer of chitosan film, and the addition of exopolymeric material to the chitosan film significantly extended mangoes shelf life [67].

### Vitamins

Vitamins are vital chemical compounds that an organism requires in small amounts to ensure appropriate metabolic activity. Menaquinone-7, a highly bioactive homolog of vitamin K, was isolated from fermented soybean and natto, the promising *B. subtilis* strain [69]. The primary sources of menaquinone in fermented foods are produced by bacteria, such as those used in the production of natto, cheonggukjang, and various cheeses [70]. Researchers have subsequently improved the production of vitamin K by employing mutagenesis and screening techniques, optimizing culture conditions, and facilitating the secretion of the desired product [71]. Mohammed and his colleagues developed a fermentation method for *B. megaterium* to enhance the production of vitamin B_12_ by providing essential supplements and dividing the fermentation process into three separate stages with different approaches. These strategies significantly improved vitamin B_12_ production up to 204.46 μg l^-1^, indicating that *B. megaterium* could be a promising candidate for large-scale vitamin B_12_ synthesis [72]. Moreover, a genetically modified strain achieved a maximum riboflavin concentration in fed-batch fermentation, exceeding the yield of *B. subtilis* RF1 [6]. Metabolic engineering is a practical approach for enhancing riboflavin production and decreasing industrial costs.

### Antimicrobial Agents and Metabolites

*Bacillus* species are extensively used in food and fermentation due to their broad-spectrum antibacterial and antifungal properties. Several *Bacillus* species biosynthesize commercially important antimicrobial compounds, including bacteriocins, lipopeptides, and antibiotics that can inhibit the proliferation of harmful bacteria, fungi, and other microbes. Bacteriocins are bioactive peptides produced by bacteria that can inhibit the growth of pathogenic bacterial species. In addition, probiotic strain *B. subtilis* produces cyclic lipopeptides, enzymes, polypeptides, and non-peptide products, which produce bacillomycin and locillomycins [73, 74]. The antimicrobial agents and metabolites exhibit efficacies against foodborne pathogens, such as *Listeria monocytogenes*, *Staphylococcus aureus*, and *B. cereus*. However, other metabolites, such as lipopeptides including surfactin, fengycin, and iturin, are synthesized by different species of *Bacillus*. Surfactin is well-known for its surface-active properties, which enable it to rupture the cell membrane of bacteria and fungi. Iturin exhibits substantial antifungal properties, effectively reducing mold deterioration [75]. Recent genomic and metabolomics approaches can be utilized to examine *Bacillus* species as a potential source of antibacterial compounds. According to Avalon *et al*. (2022), integrating genomic and metabolomics approaches effectively discovers new drugs [76]. Ong *et al*.(2019) identified a novel anti-quorum-sensing compound, demonstrating that cell density influences microbial gene expression through quorum sensing or cell-to-cell communication [77]. *Bacillus* metabolites, such as stigmatellin Y, are believed to inhibit the formation of biofilms by *P. aeruginosa* by competing with the *Pseudomonas* quinolone signaling receptor [78]. Vadakedath *et al*. (2019) provide a comprehensive analysis of this antimicrobial peptide purification process, characteristics, and mode of action from *B. licheniformis* MCC 2016, highlighting its potential use in the food industries [79]. Although a previous genome study of the probiotic strain *B. licheniformis*, isolated from naturally fermented congee in China, revealed two gene clusters related to peptide biosynthesis [80]. Lipopeptide surfactants produced from *B. licheniformis* exhibit antibacterial properties and have been employed in food preservation applications. According to Nithya *et al*. (2013), *B. licheniformis* Me1 can be utilized as a packing film and has been shown to effectively inhibit the growth of bacteria populations, increasing the shelf life of food products [81]. Notably, *B. altitudinis* and *B. licheniformis* species exhibited antagonistic activity against foodborne pathogens, such as *L. monocytogenes*, on agricultural products or in various food processing applications [82, 83]. Antibacterial polyketide metabolites isolated from seaweed-associated *B. amyloliquefaciens* exhibit a promising antibacterial spectrum against food-pathogenic microorganisms [84]. Earlier studies have demonstrated that *Bacillus* species obtained from raw honey can synthesize a diverse range of secondary compounds, effectively inhibiting the growth of foodborne pathogens. *B. subtilis* H215 was isolated from raw honey and exhibited inhibitory effects against the pathogenic fungus *Byssochlamys fulva* H25. Bacillomycin F, a purified chemical derived from *B. subtilis*, has been found to exhibit inhibitory activity against *B. fulva* [85]. Besides, the partially purified antimicrobial compounds exhibited significant antibacterial activity [86]. At the same time, iturin A, a lipopeptide exhibiting broad-spectrum antifungal activity, was isolated from *Bacillus velezensis* [87]. Notably, *Bacillus* species have high efficacy and sustainability as pest controls in agricultural and other environments. The Environmental Protection Agency (EPA) has approved certain strains of *B. subtilis*, *B. thuringiensis*, and *B. amyloliquefaciens* to be used as commercial biopesticides [88].

### Flavoring Agents

*Bacillus* species are widely recognized for their antibacterial ability to synthesize a diverse range of compounds that enhance the taste and fragrance of food products. *Bacillus* species play a crucial role in producing numerous fermented foods, significantly contributing to their taste and nutritional value [89]. These bacterial species enhance the culinary qualities of this food by creating a range of enzymes and bioactive chemicals during fermentation, which contribute to the development of complex and pleasant flavors. Due to their inherent safety and fermentability, these substances are highly prized for creating innovative and delectable food items in the modern food industry [90]. In recent years, flavoring compounds have played a crucial role in the food industry, enhancing the taste, aroma, and overall sensory experience of food products. Natural flavoring agents are extracted from natural sources and used directly in the food and feed industry. However, *Bacillus*-fermented products flavoring agents can improve the quality and safety of food products. The utilization of biological systems for pyrazine production is increasingly relevant in the food sector due to consumer preference for natural ingredients. The relationship of microorganisms, substrates, and processing conditions can lead to notable variations in flavor and texture of Dawadawa and similar fermented products, such as natto, kinema, and thua-nao [90]. *B. subtilis* strains isolated from natto can synthesize a broader range of alkyl pyrazines isolated from fermented foods [1]. Notably, the flavor of Dawadawa is attributed to its high content of amino acids, primarily glutamate, peptides, and volatile compounds that contribute to its distinctive flavor. Heat can convert the amino acid and fatty acid constituents into volatile compounds during fermentation. *Bacillus* species generate aldehydes, ketones, and acids as volatile aromas while fermenting soybeans [91].

A recent study demonstrated that the use of *Trichoderma harzianum* and *B. thuringiensis* resulted in an enhanced accumulation of volatile chemicals in lemon fruits, leading to improved aroma and quality. The aroma of fruit is influenced by the fertility of the soil and the presence of beneficial bacteria [92]. The flavor of vanilla originates from vanillin and other volatile compounds produced during the curing process, characterized by sweet aromas. Consequently, *Bacillus* colonization facilitates glucovanillin hydrolysis and vanillin synthesis during conventional curing [93]. Therefore, specific β-D-glucosidase-producing *Bacillus* species can enhance vanillin output without adverse sensory effects. Conversely, pyrazines are well-known for their distinctive nutty, roasted, and earthy aromas and are widely used in the food industry as flavor additives.

## Food Toxicity Determination

**Fig. 2:**
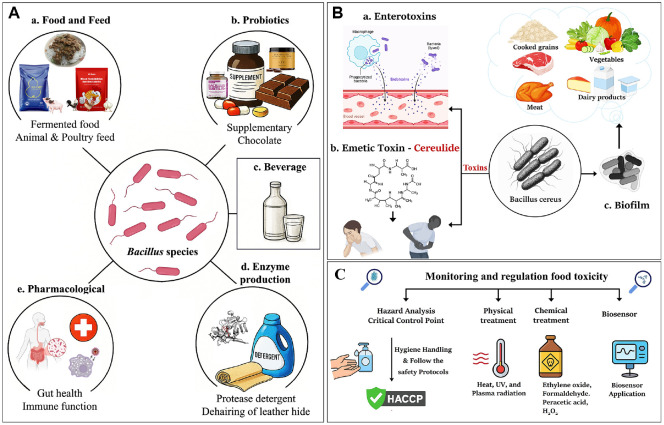
*Bacillus* species food industry application and monitoring & regulating food toxicity. (**A**) The role of *Bacillus* species in the food and pharmaceutical industry and their applications. a) Food and Feed application: Soybean fermented food, commercial animal and poultry feed (piglets, rooster, and hen). b) Probiotic application: *Bacillus* spore probiotics, prebiotics, supplementary and probiotic chocolate. c) Beverages. d) Industrial application (Protease): Detergent and dehairing of leather hide. e) Pharmacological application: *Bacillus* probiotics activity gut health, Primary immune response (macrophage, T and B lymphocyte response against antigen). (**B**) *Bacillus cereus* food toxicity and monitoring & regulating food toxicity in food and fermentation industry. a) Enterotoxin (Diarrheal type): *B. cereus* produces protein complexes that damage intestinal cells by forming pores in their membranes. This results in watery diarrhea, abdominal cramps, and nausea appearing after eating contaminated food. b) Emetic Toxin (Cereulide): This is a heat- and acid-stable cyclic peptide, disrupting mitochondrial function. It is usually performed in starchy foods like rice or pasta and causes rapid-onset nausea and vomiting. c) Biofilm Formation: *B. cereus* forms biofilms composed of extracellular polymers, proteins, and DNA. Biofilms are a significant source of food contamination across grains, vegetables, meat, and dairy. (**C**) Monitoring and regulating food toxicity in the food industry: Hygiene protocols, physical and chemical control, and biosensor application.

*Bacillus cereus* produces enterotoxins and emetic toxins (cereulide), which induce emetic and diarrheal foodborne disorders. The strain releases heat-resistant proteins known as enterotoxins, which are associated with causing diarrhea. *Bacillus cereus* can lead to food poisoning, vomiting, intense diarrhea, and meningitis, which can be lethal for patients [95] Fig. 2B (a – b). The severity of these disorders can be influenced by human factors, including age, immunological condition, and diet, as well as bacterial traits such as toxin genes and expression, and dietary composition. The minimal infective dose for *B. cereus* to induce illness is generally greater than 10^5^ CFU [94]. This bacterium is widely distributed and can readily contaminate any food production pathway. It prefers to flourish in cooked grains, meats, vegetables, and dairy products [94]. To mitigate these risks, the food industry implements various control measures, including strict hygiene protocols, monitoring of bacterial cell counts, and the use of predictive models that take into account pH and water activity. One major challenge is biofilm formation, which can be addressed through physical, chemical, and biological intervention [97, 98], as illustrated in Fig. 2B(c).

Understanding extrinsic factors that affect toxin generation and food safety, such as essential control points, is crucial. This understanding can improve prevention and Hazard Analysis Critical Control Point (HACCP) systems. Physical treatments such as heat, ultraviolet (UV) light, and plasma are effective in reducing microbial loads on food contact surfaces and equipment. Additionally, chemical agents like ethylene oxide, formaldehyde, peracetic acid, and hydrogen peroxide (H_2_O_2_) are commonly used for surface sterilization and decontamination of the processing environment (Fig. 2C). However, sterilizing packaging materials with ethylene oxide and formaldehyde are carcinogenic and unsuitable for aseptic processing. Arreola *et al*. (2019) developed a sensor array to detect gaseous H_2_O_2_ and determine the viability of spores [99]. Furthermore, biosensor technologies allow the rapid, on-site detection of *B. cereus* cells and toxins, allowing for timely interventions to prevent spoilage or outbreaks [96]. Together, these approaches enhance product quality, ensure consumer safety, and help meet the standards of HACCP and other regulatory frameworks in food fermentation processes (Fig. 2C).

## Current Global Status

Probiotics and fermented food products have diverse applications in digestive diseases, preventive healthcare, cosmetics, pharmaceuticals, and nutritional supplements, driving the markets expansion and presenting a wide range of opportunities. This growth is driven by increasing consumer interest in natural and functional foods, an aging population, the rise of e-commerce platforms, greater awareness of gut health and digestion, and rising disposable incomes. Key trends to watch include personalized probiotics, probiotic beverages, microbiome-based medicines, sustainability, and eco-friendly packaging. Recently, *Bacillus* probiotics in animal feed have been gaining popularity due to their unique biological features and evolving regulatory landscape. Asia-Pacific is the most significant and fastest-growing market for poultry and aquaculture. Aquaculture intensively studies *Bacillus* and non-LAB probiotics to reduce illness, enhance digestion, and improve water quality. Current market studies rank North America and Europe as the top markets for *Bacillus* probiotics.At the same time, the Asia-Pacific, notably China and India, is the fastest-growing area for feed and aquaculture probiotics.

Notably, *B. subtilis* is appropriate for commercial enzyme production. Incomplete data suggest that *B. subtilis* enzymes constitute around 50% of the enzyme market [101]. Enzymes are crucial in the food, feed, detergent, textile, leather, paper, and pharmaceutical sectors. In addition, microbial consortia play a vital role in baijiu (Chinese liquor) fermentation. Notably, *Bacillus* species significantly enhance product quality and flavor. Chinese liquor is known globally for its cultural and historical significance. Recently, it has increased its market share, producing 7,156,000 kiloliters and earning 170.194 billion Yuan in 2021 [101]. Japan and South Korea pioneered the use of *B. subtilis* probiotics in functional foods and supplements. The inclusion of *B. subtilis* in health products has boosted the probiotic market in China and India, demonstrating its potential in these regions. Functional foods and nutritional supplements containing *B. subtilis* are available in the US, Canada, and Europe. The market competition is moderate, with industry executives such as Bayer, BASF (Germany), and Quinlan Jocanima (Philippines) holding approximately 55% market share. China is the largest consumer, accounting for nearly 33%of sales revenue, followed by North America, with a market share of 26% [102].

## Limitations of Using *Bacillus* Species in the Food Industry

Current challenges in using *Bacillus* species for food fermentation include their heat resistance, potential contamination with genetically modified bacteria carrying antimicrobial resistance genes, and the need for improved control of spore-forming bacteria. *Bacillus* species, such as *B. cereus*, have varying heat resistance levels that impact food safety and quality [103]. Additionally, unauthorized genetically modified *Bacillus* species have been found in commercial fermentation products, raising concerns regarding antimicrobial resistance gene transfer [104]. The industrial application of *Bacillus* fermentation may encounter challenges related to environmental factors, media, and metabolite yield. Furthermore, unfavorable metabolites are generated by the strains. Using substandard ingredients, inadequate hygienic measures during production, and the absence of safety standards can compromise food safety. The lack of sufficient resources and expertise in identifying strains, their pathogenicity, and hazardous substances leads to significant complications in human health. Partially purified and uncharacterized metabolites refer to alterations in the sample that are not physiologically relevant and occur due to sample processing. Therefore, identifying specific bioactive metabolites in these species is now limited to just a few studies, and our understanding of the molecular pathways involved remains incomplete. Challenges encountered in utilizing *Bacillus* species for food fermentation include optimizing production efficiency and navigating regulatory limitations. Solutions involve genetic modifications and systems biology approaches to enhance overall performance. *Bacillus* species used for food fermentation pose challenges due to their sensitivity to antibiotics and varying levels of antioxidant activity. Selecting strains with resistance and high antioxidant potential can effectively address these challenges and enhance their applicability. Regulations are crucial in using *Bacillus* probiotic strains from different countries in human applications. However, the potential of functional foods made from *Bacillus* species in the nutraceuticals market still needs to be explored due to the ongoing debate over their probiotic or pathogen status. A critical factor in resolving this debate and gaining market acceptance is a thorough understanding of the phenotypic and genotypic features of selectively chosen *Bacillus* species. This knowledge could substantially enhance their commercial utilization of these *Bacillus* products, thereby enabling them to gain market dominance.

## Conclusion and Future Prospects

The review study examines the application of *Bacillus* species in food fermentation, with a particular emphasis on their ability to enhance nutritional value and promote the health of both humans and animals. Utilizing the most advanced and innovative techniques to analyze the microbial diversity in traditional fermented foods containing probiotic species, such as third-generation sequencing technology, can improve our understanding of the safety and beneficial qualities of probiotic *Bacillus* species. Moreover, a deeper understanding of natural fermentation processes and starter cultures could lead to future studies aimed at overcoming challenges in soybean consumption. This could result in improved product safety, quality, nutrition, and health benefits while considering economic and sustainable aspects. Additionally, it could stimulate innovation in product development. Studying how taste substances are created when probiotic *Bacillus* species collaborate in the fermentation of foods can enable more accurate regulation of the brewing process, leading to higher-quality and more delicious foods. Meanwhile, *B. subtilis* is already found in many commercial food products and is expected to play a significant role in future packaged food choices. Its potential applications extend beyond human consumption, as it is also employed in animal probiotics. Future inquiries could examine the possible advantages of *B. subtilis* in different domains, such as dermatology or dental hygiene. The crucial aspect of *Bacillus* industrial production of lipopeptides is their emulsifying, antimicrobial, and surfactant characteristics. *Bacillus*-derived lipopeptides have practical uses in biotechnology, medicine, food, and cosmetics. These compounds can effectively and comprehensively address current and emerging plant diseases, offering a flexible framework. The increasing emphasis on environmentally friendly chemicals is prompting the development of a potential new biopesticide market. Various omics technologies, including genomics, proteomics, and metabolomics, are being utilized to gain a deeper understanding of how probiotic *Bacillus* species influence the microbial composition and complex metabolic processes in natural food fermentation. Advancements in gene-editing technology have made it possible to edit individual and multiple genes, substitute gene clusters, and recombine genomes. This has dramatically enhanced our understanding of how metabolic networks are regulated and biosynthetic pathways function. Recently, scientists have successfully altered the genetic composition of various organisms using gene-editing methods, with *Bacillus* species emerging as a desirable candidate for further study.

## Figures and Tables

**Table 1:** The diversity of *Bacillus* species from indigenous commercial food.

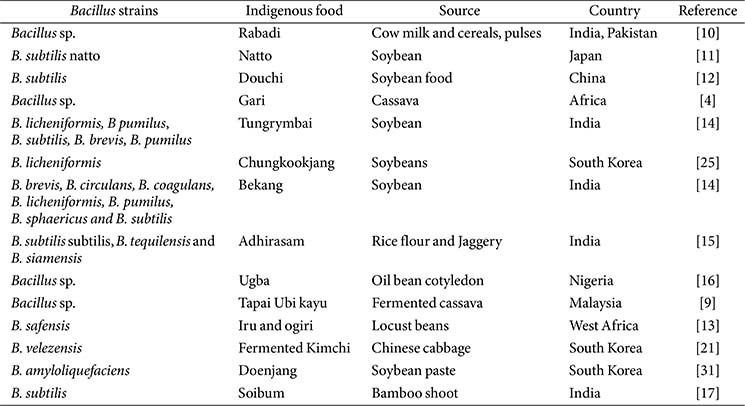

**Table 2:** Different probiotic *Bacillus* species and its health benefits.

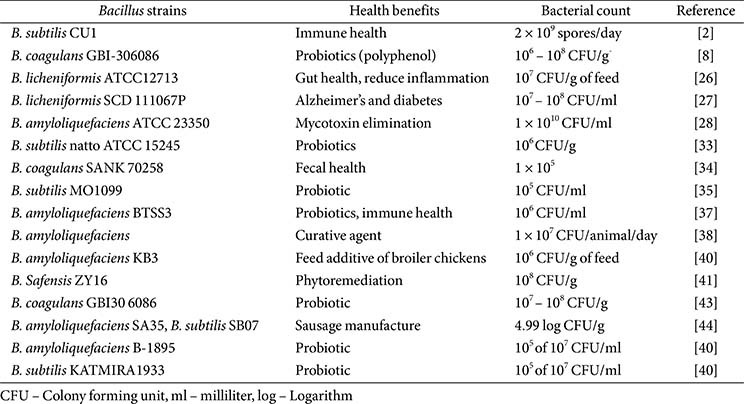

**Table 3:** Functional application of *Bacillus* metabolites in food processing and preservation.

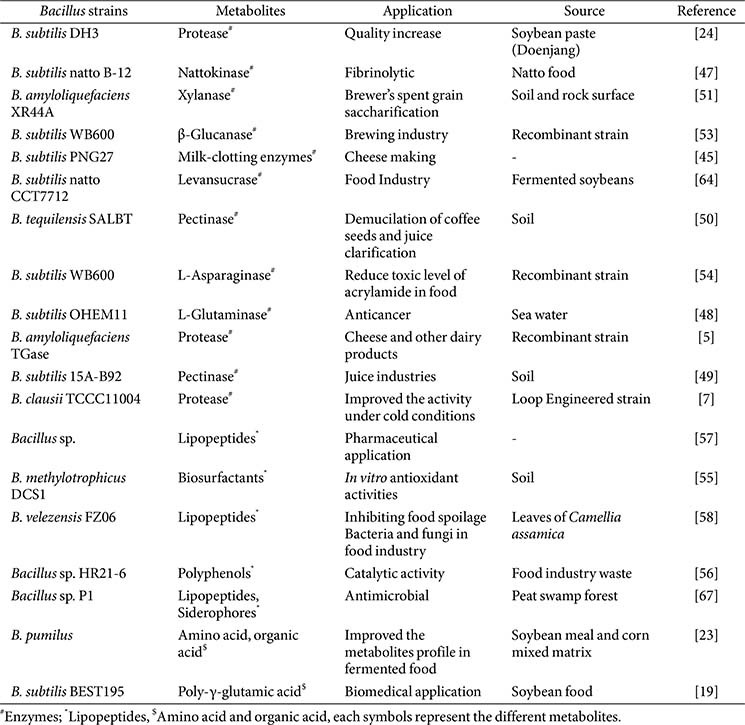

**Table 4:** Nutraceutical industry application of secondary metabolites from *Bacillus* spp.